# Anatomical variations of the coeliac trunk: a scoping review of classification systems

**DOI:** 10.1007/s11845-026-04490-0

**Published:** 2026-06-20

**Authors:** Donovan Campbell, Quitterie Lamort de Gail, Andrew D Spence

**Affiliations:** 1 School of Medicine, Dentistry and Biomedical Sciences, Queen’s University Belfast, Belfast, BT9 7BL Northern Ireland; 2 Centre for Medical Education, Queen’s University Belfast, Belfast, BT9 7BL Northern Ireland; 3 Department of Gastroenterology, Belfast Health and Social Care Trust, Belfast, BT12 6BA Northern Ireland

**Keywords:** Coeliac trunk, Anatomic variation, Cadaver, Classification, Imaging study

## Abstract

Anatomical variation of the coeliac trunk (CT) has been widely documented and carries significant clinical implications. Numerous classification systems have been proposed to describe these variations; however, inconsistency in terminology and clinical focus limit their comparability and routine application. A comprehensive synthesis of these classification frameworks is required to clarify their evolution and identify barriers to standardisation. A scoping review was undertaken in accordance with PRISMA-ScR guidance and the Joanna Briggs Institute methodology. Eligibility included studies that proposed or modified classification systems of CT anatomy using cadaveric, imaging, or combined approaches. Data extraction focused on study characteristics, classification structure, intended clinical application, and reported strengths and limitations. The findings were analysed using descriptive mapping and comparative narrative synthesis. Twenty-one studies published between 1904 and 2025 met eligibility, including cadaveric (*n*=8), exclusively radiological, (*n*=7) systematic reviews (*n*=3) and undefined (*n*=3). Frameworks increasingly prioritised surgical applicability by reducing the number of variant categories. Latter multidetector computed tomography–based studies analysed larger populations and incorporated morphometric parameters yet demonstrated substantial variability in nomenclature and criteria used to define identical branching patterns. Subsequently, anatomically similar configurations were frequently classified differently. Despite this heterogeneity, most systems described a limited number of recurring CT branching arrangements. Existing CT classification systems reflect evolving anatomical knowledge and technological advances but remain fragmented and inconsistent. The findings highlight the need for a unified, imaging-validated classification grounded in embryological principles and supported by large, global, multicentre datasets. Such standardisation would enhance diagnostic clarity, procedural safety, and interdisciplinary communication.

## Key messages

Coeliac trunk classifications have evolved from detailed embryological typologies to imaging-based clinical frameworks, but heterogeneity in terminology and structure persists.

Diagnostic and procedural interpretation remain limited by inconsistent definitions and lack of a unified, imaging-validated system.

Standardisation and multidisciplinary consensus are essential to improve reproducibility, surgical safety, and anatomical education.

## Introduction

The coeliac trunk (CT), also known as the coeliac axis or coeliac artery, is the first unpaired branch of the abdominal aorta, arising most often at the T12–L1 level [1]. It supplies essential foregut structures: the lower oesophagus, stomach, proximal duodenum, spleen, pancreas, liver, and gallbladder [1–3]. In its textbook form, the CT is a short trunk that trifurcates into the common hepatic, splenic, and left gastric arteries, a configuration famously dubbed the “Tripus Halleri” [4]. Extensive collateral networks link these branches with one another and with the superior and inferior mesenteric systems, reflecting the critical functions of these organs in digestion, metabolism, and immune defence [5–7].

Despite this canonical pattern, the CT is notorious for its variability. Bifurcations, such as hepatosplenic or gastrosplenic trunks, appear in roughly 10% of individuals [8], while rare anomalies are estimated at under 1%, including absent CT [9], coeliacomesenteric trunks (CMT) [10, 11], or accessory branches [12]. Even more complex forms, such as quadrifurcations or pentafurcations incorporate arteries like the inferior phrenic, dorsal pancreatic, or middle colic [13]. These arise from subtle deviations in the embryonic development of the ventral splanchnic arteries and their interconnections [14]. Understanding these variants is far more than academic. In hepatopancreatobiliary surgery [15, 16], vascular reconstruction [17], and interventional radiology [18], unexpected anatomy can lead to compromised perfusion and procedural complications. Certain patterns also underlie specific disorders; for example median arcuate ligament syndrome [19–21] demanding bespoke surgical or endovascular strategies.

Over the last century, clinicians and anatomists have proposed numerous classification schemes, beginning with Tandler [22] and Lipshutz’s [1] early 20th-century work and evolving through cadaveric atlases and modern imaging series. Yet a single, universally accepted system remains elusive. Discrepancies in terminology, differing methodologies between dissection and radiologic studies, and the continual identification of novel subtypes all impede consensus. For practitioners who must translate these anatomical maps into real-time clinical decisions, that inconsistency can be a critical liability.

This scoping review therefore aims to map and compare the array of CT classification systems in the literature, identify areas of agreement and discordance, and highlight the gaps that must be bridged if we are to achieve a truly standardised framework for both research and patient care. A scoping review is a form of evidence synthesis designed to map the breadth and characteristics of existing literature on a topic, following the established methodological Joanna Briggs Institute (JBI) framework.

## Materials and methods

### Inclusion criteria

#### Participants

Eligible studies involved human subjects in which the CT anatomy was assessed. This included cadaveric specimens, clinical imagining and surgical reports. No demographic or geographic restrictions were applied.

### Concept

The review focused on studies that proposed new or modified classification systems for CT variations, which typically address differences in branching patterns and origins. Studies that described variations without referencing a classification system were excluded.

### Context

Studies conducted in anatomical laboratories, clinical environments, or imaging-based settings (computed tomography or magnetic resonance angiography) were considered. All study designs were eligible, including observational studies, descriptive studies, case series, and reviews, regardless of geographic location or healthcare system.

### Types of sources

Peer-reviewed and grey literature were included, such as original research articles, reviews, dissertations, and conference proceedings, provided they discussed or applied a novel classification system. No restrictions were placed on publication date or language, and non-English sources were translated where possible (Table 1).

**Table 1:** Eligibility criteria

Inclusion	Exclusion
• Human subjects • Cadaveric specimens • Patients undergoing imaging • Patients in clinical/surgical settings • Studies involving classification systems of coeliac trunk variations • Studies including branching patterns, origins, vascular relationships • All study designs included: original research, reviews, dissertations, conference proceedings • No geographical or healthcare system limitations • No date/language restrictions	• Non-human studies • Studies not assessing coeliac trunk anatomy • Studies describing variations without using or referencing a classification system (unless used for description or comparison)

### Search strategy

A three-step search strategy was developed following the JBI methodology. First, a limited search of Ovid MEDLINE and EMBASE was conducted to identify relevant articles and extract keywords and index terms. These terms informed a comprehensive search across Ovid MEDLINE, EMBASE, and Web of Science. Finally, reference lists of included studies were screened for additional eligible sources. A protocol for this scoping review was published on Open Science Framework at https://osf.io/dkc6y.

### Study selection

**Fig. 1:**
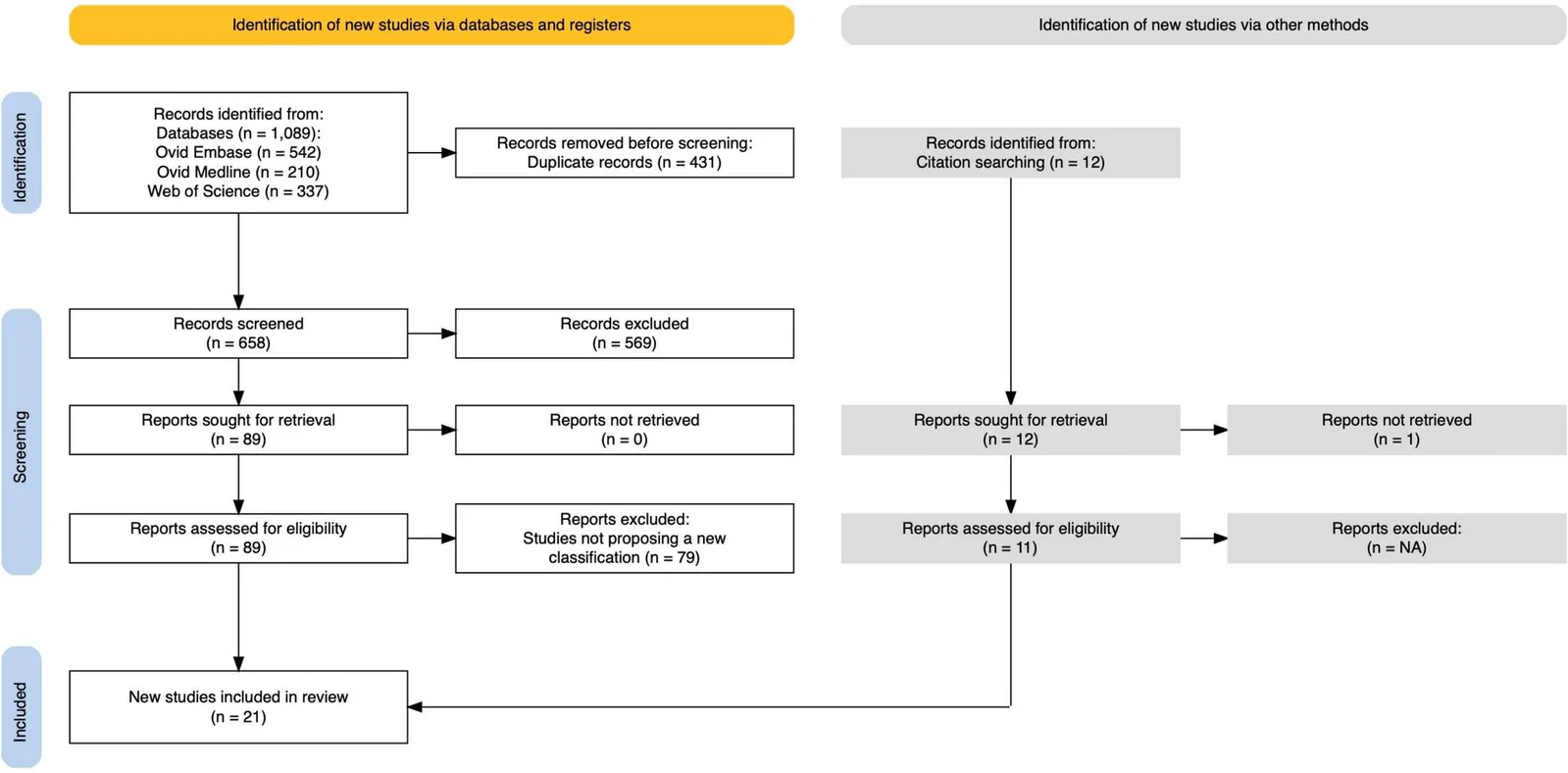
PRISMA diagram

Screening of the identified sources was conducted on Rayyan AI. Duplicates were removed, and titles and abstracts were independently screened by two researchers (QLdG and DC). Full texts of potentially relevant studies were assessed against the inclusion criteria. Conflicts were resolved through discussion with a third reviewer (ADS). As per the JBI and PRISMA-ScR guidelines, the results of the selection process are detailed in a PRISMA-ScR flow diagram (Fig. 1).

### Data extraction

Two reviewers (DC and QLdG) independently extracted data using a customised Excel spreadsheet (Microsoft Corporation, Redmond, WA, USA), developed in line with JBI methodology for scoping reviews. The tool was piloted on four studies to ensure consistency and refined as needed as per guidance from the Cochrane Handbook [23]. Extracted data included bibliographic details, study design and setting, population characteristics, classification system information, its application, key findings and commentary on strengths or limitations.

### Data analysis and presentation

Extracted data is narratively synthesised with quantitative data presented descriptively and qualitative data thematically organised. As such, there is no formal risk of bias assessment of included studies, nor critical appraisal, in keeping with the JBI methodology.

## Results

### Study selection

A total of 1,089 records were identified through database searches (Ovid Embase = 542, Ovid Medline = 210, Web of Science = 337). After removal of 431 duplicates, 658 records remained for title and abstract screening. Of these, 569 were excluded as unrelated to the review topic, leaving 89 full-text articles for retrieval and assessment. All 89 reports were successfully retrieved and reviewed for eligibility. Following full-text assessment, 79 studies were excluded because they did not propose or modify an anatomical classification system of the CT. Consequently, 10 studies met the inclusion criteria. An additional 12 records were identified through citation searching; 11 were retrieved and screened, and all met eligibility criteria, contributing to the final total of 21 included studies. The study selection process is summarised in Fig. 1.

### Characteristics of included studies

**Fig. 2:**
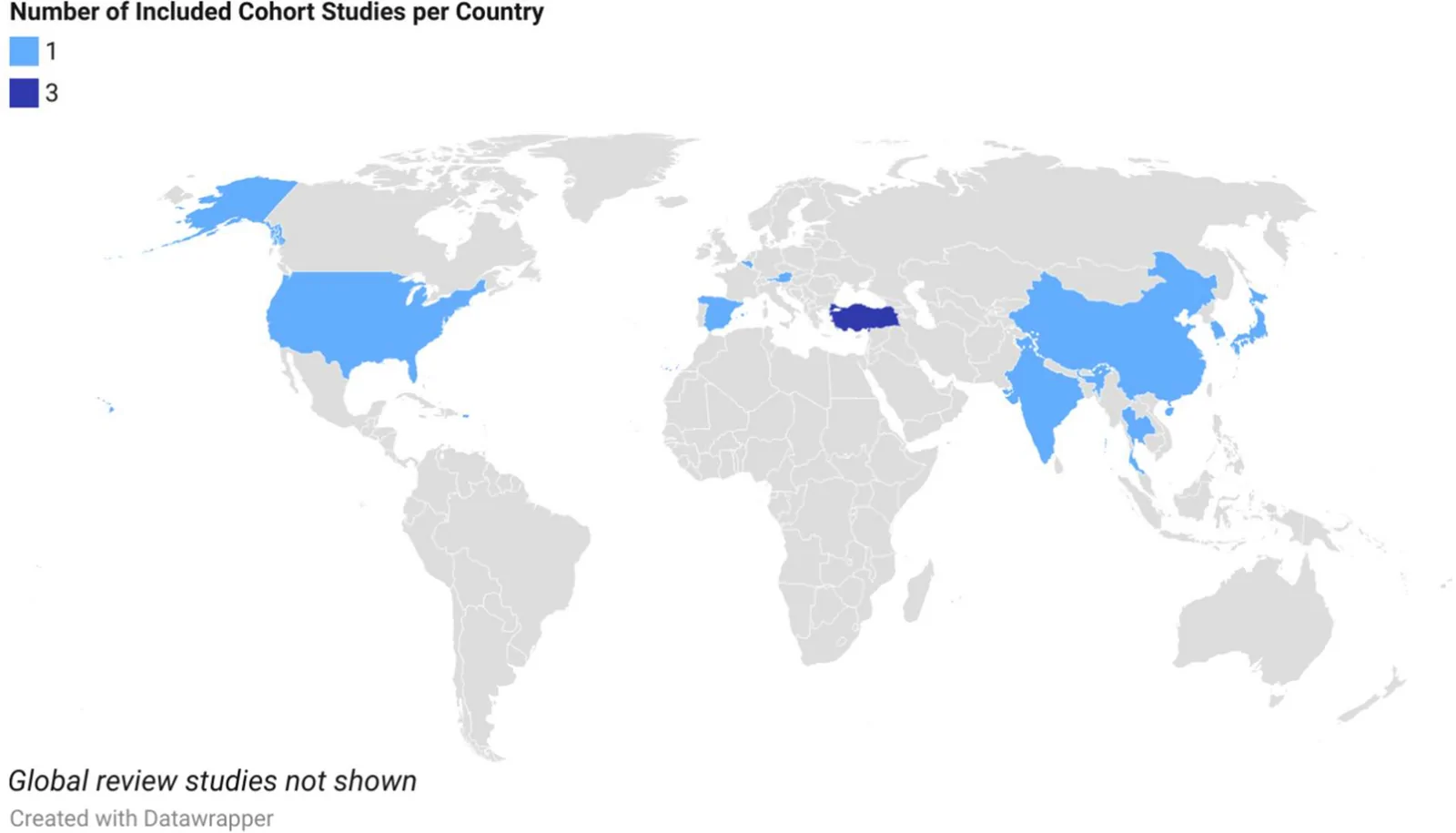
Geographic distribution of cohort studies

A total of twenty-one studies proposing classification systems of the CT and its branches were identified and are summarised in Table 2. Only studies explicitly presenting a defined classification framework were included; purely descriptive anatomical reports were excluded. Collectively, the included works illustrate the chronological and conceptual evolution of how CT anatomy has been categorised, progressing from embryological and cadaveric typologies to modern, imaging-based and clinically oriented systems. Seven of the included studies are exclusively radiological [24–30], eight are exclusively cadaveric [1, 22, 31–36], three are mixed-setting meta-analyses or systematic reviews [8, 37, 38], and three do not provide any information of the study population [39–41]. After 2007, 91% (*n* = 10/11) of studies proposing new classifications were completely or partially imaging-based, reflecting a steady methodological shift from cadaveric to radiological and multi-modality study designs. The geographic origins of the studies represent a global distribution, illustrated in Fig. 2, with the most frequently represented continents being Asia and Europe.

**Table 2:** Characteristics of Included Studies

Author(s)	Year of Publication	Country of Study	Study Design	Study Setting	Sample Size	Participant/Specimen Description
Triantafyllou et al.	2025	Global	Meta-analysis	cadaveric, radiology	34,095 (91 studies)	Adults & children. (44 studies Asia, 29 Europe, 11 North America, 5 Africa, 1 Oceania).
Turkyilmaz et al.	2023	Turkey	observational /retrospective	radiology	941	Adults. 567 males, 374 females
Mao et al.	2022	China	observational / retrospective	radiology (MDCT)	2500	Adults. 1811 males, 689 females
Acar et al.	2022	Turkey	observational / retrospective	radiology (MDCT)	340	Adults. 214 males, 126 females
Odabasioglu et al.	2021	Turkey	observational / retrospective	radiology (MDCT)	126	Adults. 66 males, 60 females
Dillibabu et al.	2021	India	observational / retrospective	radiology (MDCT)	402	Ages not specified. 254 males, 148 females
Whitley et al.	2020	Global	systematic review & meta-analysis	radiology (MDCT)	17,391 (36 studies)	Adults. (24 studies Asia, 8 Europe, 2 Africa, 2 South America).
Nateniyom et al.	2019	Thailand	observational / retrospective	cadaveric	100	Adults. 52 males, 42 females No history of abdominal surgery
Marco-Clement et al.	2016	Spain	observational / retrospective	cadaveric and radiological	639	43 cadavers (24 males, 19 females). 596 patients (430 males, 166 females)
Panagouli et al.	2013	Global	systematic review	cadaveric, surgical, radiology	12, 196	25 cadaveric studies- total 3,278 specimens; 7 radiology studies- total 6,501 patients; 3 liver transplantation studies- total 2,261 patients; 1 study combining arteriography, corrosion, and dissection, total: 156 specimens.
Song et al.	2010	South Korea	observational / retrospective	radiology (MDCT and DSA)	5002	Adults. 4015 males, 987 females
Uflacker, Renan	2007	Global	Textbook	NA	-	No demographic details.
Nelson et al.	1988	US	retrospective cohort	cadaveric	50	Ages not specified. 24 males, 26 females.
Lippert and Pabst	1985	NA	Textbook	NA	NA	No demographic details.
Vandamme et al.	1980	Belgium	observational / retrospective	arteriography, corrosion, dissection	156	No demographic details.
Michels, Aloysius	1955	Global	Textbook	cadaveric	200	No demographic details.
Morita	1935	ND	ND	ND	ND	No demographic details.
Adachi, Buntarō	1928	Japan	Textbook	cadaveric	252	No demographic details.
Eaton	1917	Global	Textbook	cadaveric	206	No demographic details.
Lipshutz	1917	NA	Textbook	cadaveric	83	No demographic details.
Tandler	1904	Austria	Textbook	cadaveric	4	One of the cadavers was a male.

Characteristics of the included studies, listed in reverse chronological order

Abbreviations: *DSA* Digital subtraction angiography, *MDCT* multi-detector computed tomography, *NA* not applicable, *ND* not described

Three systematic reviews [8, 30, 38] proposing new classification of the CT were included in our study, containing 36, 36 and 96 original studies respectively. The individual studies contained in these three global reviews are not featured in Fig. 2 as these do not propose new CT classification systems. Created with Datawrapper.

### Overview of proposed classification systems

The earliest frameworks, developed during the early twentieth century, established the foundational understanding of CT variation. Tandler [22], proposed the embryological basis of CT and mesenteric artery variations, attributing different branching patterns to the persistence or regression of ventral splanchnic arteries and their longitudinal anastomoses, however, this included only 4 cases. Eaton [32], built on the four types by introducing classes distinguishing vessel components for each type. Lipshutz [1], Morita [40] and Adachi [31] described systematic, embryologically derived classifications of the coeliac and CMTs, recognising complete absence of the CT as a distinct variant. These early anatomical studies were grounded in meticulous cadaveric dissection and sought to explain observed morphologies through persistence or regression of ventral splanchnic arteries. Closely related schemes, such as those of Michels [33] expanded this anatomical framework to encompass hepatic arterial supply, producing detailed taxonomies that remain referenced in hepatobiliary anatomy today. As summarised in Table 3, these early models prioritised descriptive completeness and embryologic reasoning, establishing the basis for subsequent refinements.

**Table 3:** Classification details

Title	Author	Year	Classification Details
Prevalence of coeliac trunk variants: A systematic review with meta-analysis.	Triantafyllou et al.	2025	Type 1 CT: Absence of the trunk, all branches originating from the abdominal aorta (AA) Type 2 CT: Bifurcation (two branches) Type 3 CT: Trifurcation (three branches) Type 4 CT: Quadrifurcation (four branches)
Evaluation of celiac artery and common hepatic artery variations by CT-angiography and new classification model.	Turkyilmaz et al.	2023	Type I Complete trifurcation Ia Classic trifurcation (Tripus Halleri) Ib Non-classic trifurcation Type II Variative trifurcation II a PHA agenesis * uncompleted CHA IIb HSpMT trunk (false trifurcation) Type III Bifurcations IIIa Hepatosplenic trunk + LGA from AA IIIb Gastrosplenic trunk IIIb1 Gastrosplenic trunk + CHA (complete or incomplete) from AA IIIb2 Gastrosplenic trunk + CHA from SMA IIIc Hepatogastric trunk + SpA from SMA IIId Double Bifurcation (LGA&RHA together from AA + RHA&SpA together from AA) Type IV Quadrifurcation LGA&SpA&GDA&acc. LHA together from AA Type V CT agenesis Coeliacomesenteric trunk LGA, SpA, CHA separately from AA SpA, LGA separately from AA + CHA from SMA
Absence of the celiac trunk: Definition, classification, multidetector computed tomography angiographic findings, and their probable embryological mechanisms	Mao et al.	2022	normal Type I (LGA + CHA + SA + SMA), Type II (HM trunk + LGA + SA), Type III (SM trunk + LGA + CHA), Type IV (GM trunk + CHA + SA), Type V (other)
Anatomical Variations of the Celiac Trunk and Hepatic Arteries with a New Classification, Based on CT Angiography.	Acar et al.	2022	Type 1: Normal trifurcated CT with typical hepatic branching pattern Type 1a: LGA arises from the CT prior to the bifurcation point of the CHA and SA Type 1b: CT trifurcates into the LGA, CHA, and SA Type 2: Normal trifurcated CT with anomalous hepatic branching pattern Type 2a: LHA arises from the PHA; replaced RHA from SMA Type 2b: RHA arises from the PHA; Replaced LHA from LGA Type 2c: PHA bifurcates into LHA and RHA; accessory LHA from LGA Type 2d: PHA bifurcates into LHA and RHA; accessory RHA from SMA Type 3: Incomplete or bifurcated CT with typical or anomalous hepatic branching pattern Type 3a: Hepatosplenic trunk; LGA from AA; (with typical hepatic branching) Type 3b: Hepatosplenic trunk; LGA from AA; RRHA from SMA Type 3c: Gastrosplenic trunk; CHA from the SMA Type 3d: Hepatogastric trunk; SA from AA (with typical hepatic branching) Type 4: No CT (typical hepatic branching) Type 5: Coeliacomesenteric trunk Common trunk for CHA, LGA, SA, and SMA
Investigation of the Coeliac Trunk Morphometry with Multidetector Computed Tomography Angiography	Odabasioglu et al.	2021	Type I Complete CT. LGA, CHA and SA present (trifurcation) Type Ia (LGA, CHA and SA are arised from common origin) Type Ib (LGA arises as the first branch) Type II Incomplete CT (bifurcation) Type IIa (Hepatosplenic trunk) Type IIb (Gastrosplenic trunk) Type IIc (Hepatogastric trunk) Type III Absence of CT (LGA, CHA and SA arise from the AA) Type IV Coeliacomesenteric trunk (CT + SMA) Type V Additional branch other than LGA, CHA and SA (quadrifurcation)
Newly Proposed Classification of Celiac Trunk Variations Based on Embryology-Correlation with Computed Tomography Angiography	Dillibabu, E, Prabakaran, M	2021	Type I – Normal trifurcation Type II(a) – Hepatosplenic trunk Type II(b) – Hepatogastric trunk Type II(c) – Gastrosplenic trunk Type III – No coeliac trunk Type IV(a) – Celiomesenteric trunk Type IV(b) – Hepatomesenteric trunk Type IV(c) – Right hepatomesenteric trunk Type IV(d) – Gastromesenteric trunk Type IV(e) – Splenomesenteric trunk Type IV(f) – Hepatosplenomesenteric trunk Type IV(g) – Gastrosplenomesenteric trunk Type V – Coeliaccolic trunk Type VI(a) – Coeliophrenic trunk (CT + CIPA) Type VI(b) – Coeliophrenic trunk (CT + RIPA) Type VI(c) – Coeliophrenic trunk (CT + LIPA) Type VI(d) – Coeliophrenic trunk (CT + RIPA + LIPA) Others – Additional branches
Variations of the celiac trunk investigated by multidetector computed tomography: Systematic review and meta-analysis with clinical correlations	Whitley et al.	2020	1. Complete coeliac trunk (classical) 2. Incomplete coeliac trunk 2a. Gastrosplenic trunk (GST) / Splenogastric trunk (SGT) 2b. Hepatosplenic trunk (HST) 2c. Hepatogastric trunk (HGT) 3. Absence of the coeliac trunk – no common trunk 4. Complete coeliacomesenteric trunk 5. Incomplete coeliacomesenteric trunk 5a. Hepatosplenomesenteric trunk (HSMT) 5b. Gastrosplenomesenteric trunk (GSMT) 6. Celiacomesenteric anastomosis (Arc of Buhler) 7. Cases not described (incomplete data, no consensus)
Anatomy of the vasculature supplying hepatobiliary structures and celiac trunk branching patterns in the Thai population	Nateniyom et al.	2019	Type I: SA + CHA (LGA from aorta) Type II: LGA + SA + CHA (classic) Type III: LGA + SA + DPA (CHA from SMA) Type IV: SA + CHA + LtIP (LGA from aorta) Type V: LGA + SA + CHA + rRHA Type VI: LGA + SA + CHA + DPA Type VII: LGA + SA + CHA + SGA Type VIII: LGA + SA + CHA + LtIP Type IX: LGA + SA + CHA + common trunk of LtIP and RtIP Type X: LGA + SA + CHA + RtIP Type XI: LGA + SA + CHA + LtIP + RtIP Type XII: LGA + SA + CHA + rRHA + RtIP Type XIII: LGA + SA + CHA + rRHA + LtIP Type XIV: LGA + SA + CHA + DPA + LtIP
Anatomical variations of the celiac trunk: cadaveric and radiological study.	Marco-Clement et al.	2016	Type I: Complete coeliac trunk – LGA, SA, and CHA Type Ia: Complete bifurcated coeliac trunk (LGA arises first) Type Ib: Complete trifurcated coeliac trunk Type Ic: Complete coeliac trunk with extra-branches Type II: Incomplete coeliac trunk Type IIa: Hepatosplenic trunk Type IIb: Gastrosplenic trunk Type IIc: Hepatogastric trunk Type III: Absence of the coeliac trunk -didn’t actually observe this in the study. Type IV: Coeliacomesenteric trunk - didn’t actually observe this in the study.
Variations in the anatomy of the celiac trunk: A systematic review and clinical implications.	Panagouli et al.	2013	Type I: Trifurcation of the CT into the LGA, CHA, and SA. Form 1: True tripod – common origin of LGA, CHA, and SA (Tripus Halleri). orm 2: False tripod – division into two branches with the third arising earlier along the CT. Form 2a: LGA is the first branch. Form 2b: CHA is the first branch. Form 2c: SA is the first branch. Type II: Bifurcation of the CT. Form 1: Hepatosplenic trunk, LGA arising from the AA. Form 2: Hepatosplenic trunk, no normal LGA. Form 3: Hepatosplenic trunk and gastromesenteric trunk. Form 4: Splenogastric trunk, CHA arising from the AA. Form 5: Splenogastric trunk, CHA arising from the SMA. Form 6: Splenogastric trunk and hepatomesenteric trunk. Form 7: Hepatogastric trunk, SA arising from the AA. Form 8: Hepatogastric trunk, SA arising from the SMA. Form 9: Hepatogastric trunk and splenomesenteric trunk. Type III: Presence of additional branches from the CT. Type IV: Coeliacomesenteric trunk – common origin of the CT and SMA. Type V: Variations in the origin of the CHA. Type VI: Hepatosplenomesenteric trunk – common origin of the CHA, SA, and SMA, with the LGA originating independently or as a branch of the others. Type VII: Absence of the CT – LGA, CHA, and SA arise independently. Type VIII: Splenogastromesenteric trunk – common origin of the LGA, SA, and SMA, with CHA originating independently or as a branch of the others. Type IX: Splenogastric trunk giving rise to a common inferior phrenic trunk. Type X: Coeliac-bimesenteric trunk – common origin of the CT, SMA, and IMA.
Celiac Axis and Common Hepatic Artery Variations in 5002 Patients: Systematic Analysis with Spiral CT and DSA	Song et al.	2010	Normal anatomy * 4457 (89.1%) Anatomic variation: 482 (9.6%) HSp trunk + LG + SM1: 221 (4.42%) HM trunk + GSp trunk: 132 (2.64%) Coeliacomesenteric trunk 53: (1.06%) HSpM trunk + LG 34: (0.68%) HM trunk + LG + Sp 12: (0.24%) CHA + GSp trunk + SM: 11 (0.22%) HG trunk + SpM trunk: 8 (0.16%) CHA + LG + Sp + SM: 5 (0.10%) CHA + GSpM trunk: 3 (0.06%) CHA + LG + SpM trunk: 1 (0.02%) HG trunk + Sp + SM: 1 (0.02%) HSp trunk + GM trunk: 1 (0.02%) HGM trunk + Sp: 0 CHA + GM trunk + Sp 0 Ambiguous CT anatomy 63 (1.26%) 1) persistent anastomotic channels 2) absent CHA because separate origins of heaptic and gastroduodenal aas.
Atlas of vascular anatomy: an angiographic approach	Uflacker	2007	Type I: Hepato-gastro-splenic Trunk Classic. Type 1a) Hepatosplenic trunk. Here the LGA arises from the trunk. Type 1b) Hepatogastrosplenic trunk. The classical trifurcation. Type 1c) Gastrosplenic trunk. The splenic is dominant and the hepatic arises from the splenic. Type II: Hepatosplenic Trunk Type III: Hepatogastric Trunk Type IV: Hepato-splenic-mesenteric Trunk Type V: Gastrosplenic Trunk The middle hepatic artery arises from the SMA or aorta. If absent is replaced by the right, left or both. Type VI: Coeliac-mesenteric Trunk SMA arises from trunk. <1%. Type VII: Celiac-Colic Trunk Middle/left arise from CT. Type VIII: Absent Coeliac Trunk
Anatomic variants of the celiac, superior mesenteric, and inferior mesenteric arteries and their clinical relevance	Nelson et al.	1988	Focuses on origins of each vessel rather than branches of trunk. Classic Coeliac trunk (60%) Coelicacomesenteric trunk (2%) CHA originating from SMA (8%)
Arterial Variations in Man: Classifcation and Frequency	Lippert, Pabst	1985	1: Complete coeliac trunk (gastrohepatolienal trunk) 84% 1a: Normal tripus Halleri (25%) 1b: Early LGA origin (49%) 1c: Fourth branch: additional dorsal pancreatic artery 2. Incomplete coeliac trunk 9% 2a: Hepatosplenic trunk (5%) 2b. Gastrosplenic trunk (3%) 2c. Gastrohepatic trunk (1%) 2c. Absent CT (< 1%) 3. Common CT and SMA origins(7%) 3a. Gastrohepatosplenomesenteric trunk (2%) 3b. Hepatosplenomesenteric trunk (1%) 3c. Hepatomesenteric trunk (3%) 3d. Splenomesenteric trunk (< 1%)
The Branches of the Celiac Trunk	J. P. J. Vandamme; J. Bonte	1985	The three classical branches: Only two of the classical branches: (1) Truncus gastrolienalis (CHA from SMA or AA) (2) Truncus hepatolienalis (LGA from AA) (3) truncus hepatogastricus (splenic from SMA) Absence of CT Truncus coeliacomesentericus
Blood supply and anatomy of the upper abdominal organs, with a descriptive atlas	Michels	1955	Type I: Hepatolienogastric Trunk LGA + lienal (splenic) and hepatic A. It occurred in 89% of cases. In 64.5% of cases, it was complete. In addition, it gave rise to the dorsal pancreatic or a colic branch (middle/left/accessory). In 11.5% there was a hetpatomesenteric trunk (SMA). Tripod of Haller occurred in 25% of cases. Type II: Hepatolienal Trunk 3.5% of cases. The LGA arises directly from the AA, splenic or hepatic artery. May be accompanied by a hepatomesenteric or hepatogastric trunk. Type III: Hepatolienomesenteric Trunk 0.5% of cases. The hepatic, lienal and SMA arise from a common origin. LGA from AA. Type IV: Hepatogastric trunk 1.5% of cases. LGA and hepatic arise together. The lienal from the SMA forming a hepatolienal trunk. If the hepatolienal trunk gives off a hepatic it is modified to the hepatolienomesenteric. (Type III) Type V: Lienogastric Trunk 5.5% of cases. The lienal and the LGA arise from a common trunk and the hepatic is replaced by another source. Aorta (2%), SMA (2.5%). A lienogastric trunk may give rise to a gastroduodenal, the latter replaced from the hepatic. It may also give rise to an aberrant left hepatic. Type VI: Coeliacomesenteric Trunk CHA, LGA, S, SMA arise together. Type VII: Coeliacocolic Trunk The middle or left colic from CT. Concomitantly the dorsal pancreatic supplies the large intestine. In some cases, the coeliacal dorsal pancreatic functions as an accessory middle colic. It crucially can anastomose with the SMA, middle colic and IMA.
Reports and conception of three anomalous cases on the area of the celiac and the superior mesenteric arteries	Morita	1935	Coeliac Trunk Variants Type I – Hepatogastrosplenic trunk (Classical type) Common trunk origin of LGA, SA, and CHA. Type II – Hepatosplenic trunk Common trunk origin of CHA and SA, with LGA directly from the AA. Type III – Gastrosplenic trunk Common trunk origin of LGA and SA, with CHA directly from the AA. Type IV – Hepatogastric trunk Common trunk origin of CHA and LGA, with SA directly from the AA. Type V – Absent CT Independent origin of LGA, SA, and CHA directly from the AA. Coeliacomesenteric Trunk Variants Type I′ – Coeliacomesenteric trunk (Classical type) Common trunk origin of coeliac and superior mesenteric arteries, dividing into CT and SMA; CA further divides into LGA, SA, and CHA. Type II – Common trunk of any two CT branches with SMA, third from aorta - Type II′ – Hepatosplenomesenteric trunk (HSMT): CHA, SA, SMA common origin; LGA separately from AA. - Type II″ – Hepatogastromesenteric trunk (HGMT): CHA, LGA, SMA common origin; SA separately from AA. - Type II‴ – Gastrosplenomesenteric trunk (GSMT): LGA, SA, SMA common origin; CHA separately from AA. Type III – Common trunk of any one CT branch with SMA, other two independent - Type III′ – Hepatomesenteric trunk (HMT): CHA + SMA common origin; LGA and SA independently from AA. - Type III″ – Gastromesenteric trunk (GMT): LGA + SMA common origin; CHA and SA independently from AA. - Type III‴ – Splenomesenteric trunk (SMT): SA + SMA common origin; CHA and LGA independently from AA. Type IV – Common trunk of one CA branch with SMA, other two share common origin from AA. - Type IV′ – Hepatomesenteric trunk (HMT) + Gastrosplenic trunk (GST): CHA + SMA common origin; LGA + SA common origin from AA. - Type IV″ – Gastromesenteric trunk (GMT) + Hepatosplenic trunk (HST): LGA + SMA common origin; CHA + SA common origin from AA. - Type IV‴ – Splenomesenteric trunk (SMT) + Hepatogastric trunk (HGT): SA + SMA common origin; CHA + LGA common origin from AA.
Das arteriensystem der Japaner	Adachi, Buntarō	1928	Type I: Hepatogastrosplenic The LGA, splenic and hepatic arose from a common trunk. 87.7%. Type II: Hepatosplenic Trunk 6.3%. LGA is directly from the AA. Type III: Hepatosplenicomesenteric Trunk LGA directly off AA. Hepatic, splenic and SMA from a common trunk. Type IV: Coeliacomesenteric Trunk LGA, splenic, hepatic and SMA from a common trunk. Type V: Gastrosplenic and Hepatomesenteric 0.4%. The LGA and the splenic form a common trunk directly from AA. The hepatic and SMA arise together. Type VI: Gastrosplenic 2%. The hepatic A is missing. The liver is supplied by an accessory left, right or both hepatic A.
The coeliac axis	Eaton	1917	Type I: LGA and splenic form a common trunk, the hepatic from aorta or SMA. Type II: Tripod coeliac with classes showing accessory hepatics and a pancreatic branch. Type III: Common trunk having the left gastric as its first branch with classes showing accessory hepatics and a pancreatic branch. Type IV: LGA as a separate branch from the AA, the hepatic and the splenic forming a common trunk which may have a pancreatic branch. In 7% LGA gives off an accessory hepatic.
A Composite Study of the Coeliac Axis Artery	Lipshutz	1917	Type I: Common trunk the LGA, splenic and hepatic. 75%. Type II: Common trunk for the hepatic and splenic; left gastric from AA. 15%. Type III: Common trunk for hepatic and LGA; splenic from AA. 6%. Type IV: Common trunk for LGA and splenic; hepatic from AA. 4%.
Varietaten Der Arteria Coeliae	Tandler	1904	Type I – Coeliacomesenteric trunk (CT + SMA common origin); trifurcates into LGA, CHA, SA. Type II – Short CT–SMA trunk; CHA splits into LHA + GDA; RHA arises directly from SMA. Type III – Typical coeliacomesenteric trunk where SMA gives LGA, CHA (dividing into proper hepatic + GDA), and continues as SMA; SA arises separately. Type IV – Separate origins: short common trunk for right inferior phrenic + LGA; SA and CHA arise independently—approaching absent CT configuration.

Abbreviations: *AA* Abdominal aorta, (*acc. LHA*) accessory left hepatic artery, *CT* coeliac trunk, *CHA* common hepatic artery, *CIPA* common inferior phrenic artery, *DPA* dorsal pancreatic artery, *GDA* gastroduodenal artery, *GMT* gastromesenteric trunk, *GST* gastrosplenic trunk, *HA* hepatic artery, *HMT* hepatomesenteric trunk,  *HGT* hepatogastric trunk, *HST* hepatosplenic trunk, *HSMT* hepatosplenomesenteric trunk, *IPA* inferior phrenic artery , *LGA* left gastric artery, *LHA* left hepatic artery, *LIPA* left inferior phrenic artery, *PHA* proper hepatic artery, rRHA replaced right hepatic artery, right hepatic artery, *RIPA* right inferior phrenic artery, SGA short gastric artery , *SA* splenic artery, *SMT* splenomesenteric trunk, *SMA* superior mesenteric artery

During the mid- to late twentieth century, classifications became progressively standardised and clinically oriented, however, demographic detail or sample size was sparsely reported, and sometimes altogether omitted as seen by Uflacker [41] and Lippert and Pabst [39]. Vandamme and Bonte [36], and Nelson et al. [35] both oriented their classifications to focus on vessel origin, while later cadaveric series such as Marco-Clement et al. [37] and Nateniyom et al. [34] refined type categories with clearer definitions of hepatosplenic, gastrosplenic, and hepatogastric trunks. Panagouli et al., [38] synthesised existing anatomical classifications into a unified reference framework, demonstrating an increasing effort to reconcile disparate terminologies.

Following the widespread adoption of multidetector computed tomography (MDCT), classifications evolved toward imaging-based reproducibility and surgical applicability. As methodological rigour increased, classifications grew in size and types became more clearly defined, as seen by Song (2010)’s clarification of nomenclature around “ambiguous celiac axis anatomy”, accompanying their fifteen-type classification system. Whitley et al. [30] compiled data on reported typologies, focussing on subtypes of bifurcations, based primarily on trunk components and secondarily on origin of the replaced artery. A different systematic review, by Dillibabu et al. [25], presented an eighteen-type embryology-based classification, and expanded this with tabulation of co-existing types ranked by prevalence. Odabaşıoğlu et al. [27] proposed a morphometry-integrated model combining branching pattern analysis with vessel diameter measurements, representing an intersection between anatomical precision and radiological practicality. Türkyılmaz et al. [29] extended this concept by presenting a unified CT–common hepatic artery (CHA) classification, simplifying prior systems into five major branching types optimised for imaging interpretation. Other studies by Mao et al. [26], Acar [24], and Triantafyllou et al. [8] mirrored this trajectory, illustrating an emphasis on reproducibility, standardised nomenclature, and preoperative mapping within radiological and surgical practice.

## Discussion

### Summary of findings

This scoping review identified and synthesised the findings of twenty-one studies that proposed or modified classification systems describing variations of the CT. Collectively, these classifications demonstrate a clear chronological evolution from embryologically based cadaveric schemes to simplified, imaging-oriented frameworks designed for clinical application. Early anatomical systems, such as those of Adachi [31] and Morita [40] provided the embryologic rationale for arterial variation, while mid-century and later frameworks, including those by Michels [33], and Panagouli et al. [38] sought to consolidate and standardise terminology. More recent MDCT studies, such as those by Odabaşıoğlu et al. [27]; Whitley et al. [30]; Dillibabu et al. [25]; Türkyılmaz et al. [29] integrated morphometric data and unified coeliac–hepatic classifications to improve reproducibility and surgical relevance. Triantafyllou et al. [8] went further extracting prevalence ranges for each configuration from individual studies, and compared frequencies across cadaveric and imaging-based cohorts, but deliberately avoided pooled estimates due to heterogeneity in definitions and study design.

Despite methodological and terminological heterogeneity, all systems converge on a limited number of recurring branching patterns, highlighting both the anatomical consistency of the CT and the enduring need for a unified, imaging-validated classification applicable across clinical and research settings.

### Comparative analysis of classification features

Marked heterogeneity exists among published classification systems of the CT, reflecting both the evolution of imaging technology and the differing clinical aims of investigators. Early embryological frameworks, including those by Adachi [31] and Morita [40], were derived from small cadaveric cohorts (< 300) and sought to explain arterial variation through persistence of ventral splanchnic roots. Although anatomically precise, these descriptive schemes lack reproducibility in modern radiology. Mid-century systems, such as Michels [33] simplified earlier classifications to support hepatobiliary surgery, consolidating multiple subtypes into a limited number of reproducible categories.

With the introduction of MDCT and angiographic reconstruction, classification became increasingly data-driven and clinically oriented. Imaging studies often include several hundred to over 1000 participants, allowing quantification of trunk length, vessel calibre, and branching prevalence [26]. However, this expansion introduced new variability with reported frequencies of the classic trifurcation ranging from 9 to 75% depending on whether an early left gastric artery (LGA) origin was considered typical [8, 29]. Such inconsistency in diagnostic thresholds underscores how nomenclatural divergence, not true anatomical difference, drives variation across studies.

From a diagnostic standpoint, this heterogeneity complicates radiological interpretation and inter-observer reliability. Conventional cadaveric categories frequently fail to capture calibre asymmetry, short common trunks, or accessory arteries visible only on volumetric angiography [27, 29]. Procedurally, inconsistent classification can influence pre-operative mapping and intra-operative planning in pancreaticoduodenectomy, and selective embolisation, where variant recognition determines anastomotic strategy and perfusion adequacy [24].

When comparing strengths and limitations, the contrast between cadaveric precision and imaging generalisability becomes evident. Modern CT-based classifications achieve broader applicability and facilitate morphometric analysis, yet they risk oversimplification and occasional misclassification when small branches fall below reconstruction thresholds. Hybrid frameworks, such as those of Panagouli et al. [38] and Nateniyom et al. [34] attempt to reconcile these approaches by merging embryological rationale with radiological practicality. Despite such progress, a standard validated classification remains absent.

A major source of difficulty in comparing the included studies was the extent to which authors described the same arterial configuration using different terms, criteria, and hierarchical structures. Even widely recognised variants such as the hepatosplenic trunk or classic trifurcation were not uniformly defined. For example, a hepatosplenic configuration appears as Adachi Type II, Morita Type II, Panagouli Type II, and Marco-Clement Group 2 [31, 37, 38, 40], yet each author applies subtly different inclusion criteria regarding trunk length, the presence of accessory branches, or whether the LGA must arise independently. Similarly, the classic Tripus Halleri is labelled Adachi Type I, Morita Type I, Panagouli Type I-a/I-b, or Odabaşıoğlu Type I [27, 31, 38, 40], with these discrepancies creating the appearance of divergent anatomy when the underlying pattern is identical.

More complex variants amplify this inconsistency. The CMT is variously defined across Morita [40], Adachi [31], Panagouli et al. [38], Nateniyom et al. [34], and Acar [24], each offering different subtype structures based on embryological interpretation, radiological appearance, or morphometric thresholds. What one system classifies as a true CMT, other divides into separate “hepatomesenteric” and “splenomesenteric” categories. Gastrosplenic and hepatogastric trunks are similarly reclassified across studies depending on whether hepatic artery variants are integrated or analysed separately. Even the absence of a CT is not uniformly defined: Morita Type VI, Panagouli Type VIII, and Nateniyom Group 3 [34, 38, 40] describe comparable configurations yet differ on how many independent origins constitute “true absence.”

These inconsistencies reflect deeper methodological and conceptual differences between cadaveric and imaging-based studies, between embryological and clinical priorities, and between systems that isolate the CT and those that incorporate hepatic arterial variants. Critically, they hinder synthesis, as studies may report different “types” despite describing anatomically identical patterns.

A summary of the key types included in each classification is synthesised in Table 4.

**Table 4:** Comparison of classification typology

Author	Classic Trifurcation	Other Trifurcation	Bifurcation	Quadri-furcation	Absent CT	CMT
Triantafylloua et al.	✔		✔	✔	✔	
Turkyilmaz et al.	✔	✔	✔	✔	✔	✔
Mao et al.	✔			✔		
Acar et al.	✔		✔		✔	✔
Odabasioglu et al.	✔		✔	✔	✔	✔
Dillibabu, E, Prabakaran, M	✔		✔		✔	✔
Whitley et al.	✔		✔		✔	✔
Nateniyom et al.	✔	✔	✔	✔		
Marco-Clement et al.	✔		✔	✔	✔	✔
Panagouli et al.	✔	✔	✔		✔	✔
Song et al.	✔				✔	✔
Uflacker	✔	✔	✔		✔	
Nelson et al.	✔					✔
Lippert, Pabst	✔		✔	✔	✔	✔
J. P. J. Vandamme; J. Bonte	✔		✔		✔	✔
Michels	✔	✔	✔			✔
Morita	✔		✔		✔	✔
Adachi	✔	✔	✔			✔
Eaton	✔		✔	✔		
Lipshutz	✔		✔			
Tandler					✔	✔

Abbreviations: *CT* Coeliac trunk, *CMT* coeliacomesenteric trunk

Although individual classification systems describe CT morphology using different structures and terminology, six overarching categories can be identified across the literature. These include classic trifurcation, in which the CT divides into the LGA, splenic artery, and CHA; other trifurcation, defined as any three-branch configuration that does not conform to the classic pattern; bifurcation, where the CT divides into two principal branches; quadrifurcation, comprising any combination of four arterial branches; absence of the CT, in which the LGA, SA, and CHA arise independently from the abdominal aorta; and the CMT, where the CT and superior mesenteric artery share a common origin. Table 4 illustrates the distribution of these broad typologies across the included classification systems but does not capture the level of granularity within each category, as authors vary considerably in how extensively they subdivide these patterns. For example, Panagouli et al. [38] described nine distinct trifurcation subtypes, whereas Triantafyllou et al. [8] grouped all trifurcation configurations within a single category.

Notably, the development of CT classifications appears to have followed a cyclical trajectory. Early frameworks proposed by Tandler [22] and Lipshutz [1] adopted relatively simple descriptive models grounded in embryological principles. With the introduction of radiological studies, particularly multidetector computed tomography, classification systems became increasingly complex as additional subtypes were introduced to reflect newly visible anatomical detail. More recent classifications, exemplified by Triantafyllou et al. [8] suggest a return to simplification, consolidating variants into broader, clinically recognisable categories to balance anatomical completeness with diagnostic usability.

### Summary of gaps and limitations

Despite extensive literature describing CT anatomy, significant gaps remain in the consistency and validation of existing classification systems. Early cadaveric studies and contemporary imaging-based research represent distinct methodological eras, yet few efforts have bridged embryological theory with radiological application. The evidence base is geographically uneven, with a predominance of Asian and Mediterranean cohorts, limiting generalisability across populations. Considerable variation persists in terminology, inclusion criteria, and the number of categories used, hindering cross-study comparability and synthesis. Many proposed classifications lack reproducibility testing or interobserver reliability assessment, and no single scheme has achieved universal acceptance. As a scoping review, this work did not undertake formal quality appraisal or quantitative synthesis, and non-English or unpublished sources may have been missed. Collectively, these limitations highlight the need for a standardised, imaging-validated framework integrating developmental anatomy with clinically applicable classification of the CT and its variants.

### Future directions

Future work should prioritise the development of a unified classification system that integrates embryological findings with modern imaging capabilities. A central step will be establishing a single, consensus terminology, endorsed jointly by anatomical, radiological, surgical and interventional societies. This initiative should formalise how branching patterns, accessory vessels, and transitional configurations are defined. A practical two-tier structure may be most effective, with primary categorisation based on CT branching patterns, followed by a secondary layer describing clinically relevant hepatic arterial variants.

Robust validation frameworks will be essential. These should include interobserver agreement studies across specialties, cross-modality imaging validation, and external validation in diverse global populations. A coordinated multicentre, multi-continent imaging and clinical study would provide the scale needed to link anatomical variation to meaningful patient outcomes, including operative complexity, complication rates, and postoperative perfusion patterns. Such datasets would also mitigate current geographic biases and strengthen generalisability.

Implementation strategies must be considered from the outset. A harmonised classification should be adaptable to structured radiology reporting and translatable into surgical planning. With this, emerging technologies offer supplementary support. Machine-learning–assisted vascular mapping could automate preliminary labelling based on consensus definitions, augmenting expert anatomical interpretation. Educational integration is equally important wherein a standardised system could underpin anatomy curricula, simulation training, 3D and VR visualisation platforms, and cadaveric–imaging correlation modules.

Finally, future frameworks should account for pathology-related implications, including patterns relevant to median arcuate ligament syndrome, aneurysm formation, and collateral flow during arterial stenosis or oncological resection. Integration with adjacent vascular territories, such as pancreaticoduodenal arcades and SMA–coeliac collateral pathways, will ensure that classification reflects functional anatomy rather than isolated arterial segments.

### Conclusion

This scoping review mapped the evolution of CT classification systems over more than a century, demonstrating a clear progression from detailed embryological and cadaveric typologies to simplified, imaging-based frameworks designed for clinical use. Despite this advancement, substantial heterogeneity persists. The classifications reviewed converge on a core set of recurring branching patterns, yet differ in how variants are defined, named, and prioritised.

Future progress will require a unified, consensus-driven classification that integrates embryological rationale with modern imaging findings, incorporates clinically relevant hepatic variants, and is validated across populations and modalities. Multicentre global collaboration, standardised reporting, and supplementary use of computational tools will be essential to achieving this goal. Establishing such a framework would improve diagnostic clarity, enhance surgical and interventional safety, and provide a coherent anatomical foundation for future research and education.

## Appendix

**Table 5:** Search strategy

Database	Search Strategy	Number
*OVID MEDLINE*	*Ovid MEDLINE(R) ALL < 1946 to July 02*,* 2025>* *1 Celiac Artery/* *2 (coeliac axis or coeliac trunk or coeliac artery).mp.* *3 (celiac axis or celiac trunk or celiac artery).mp.* *4 Anatomic Variation/* *5 anatom* varia*.mp.* *6 1 or 2 or 3* *7 4 or 5* *8 6 and 7*	210
*WOS Core Collection*	ALL=(“c$eliac artery” or “c$eliac trunk” or “c$eliac axis”) AND ALL=( "anatom* varia*”) NOT TS=( animal* )	337
*OVID EMBASE*	Embase < 1974 to 2025 July 02> *1 Celiac Artery/* *2 (coeliac axis or coeliac trunk or coeliac artery).mp.* *3 (celiac axis or celiac trunk or celiac artery).mp.* *4 Anatomic Variation/* *5 anatom* varia*.mp.* *6 1 or 2 or 3* *7 4 or 5* *8 6 and 7* *9 limit 8 to (humans)*	542

## References

[1] Lipshutz B (1917) A Composite Study of the Coeliac Axis Artery. Ann Surg 65(2):159–169. 10.1097/00000658-191702000-00006 PMC1426316 17863663

[2] Biyikli E, Sever IH, Baltacioglu F (2022) Reliability of coeliac and superior mesenteric artery origin level in lumbosacral transitional vertebrae detection and vertebral numbering. Marmara Med J 35(1):48–52. 10.5472/marumj.1030355

[3] Rusu MC, Jianu AM, Manta BA, Hostiuc S (2021) Aortic origins of the celiac trunk and superior mesenteric artery. Diagnostics 11(6). 10.3390/diagnostics11061111 PMC8234440 34207138

[4] von Haller A (1756) Icones anatomicae in quibus aliquae partes corporis humani delineatae proponuntur et arteriarum potissimum historia continetur. Vandenhoeck. https://collections.library.utoronto.ca/view/anatomia:RBAI041

[5] Appanraj P, Mathew AP, Kandasamy D, Venugopal M (2021) CT reporting of relevant vascular variations and its implication in pancreatoduodenectomy. Abdom Radiol 46:3935–3945. 10.1007/s00261-021-02983-3 33738555

[6] Balachandran A, Darden DL, Tamm EP, Faria SC, Evans DB, Charnsangavej C (2008) Arterial variants in pancreatic adenocarcinoma. Abdom Imaging 33(2):214–221. 10.1007/s00261-007-9235-z 17435979

[7] Omar R, Kisansa M, Dehnavi AD (2021) The prevalence of anatomical variants of the coeliac trunk and renal arteries on contrast-enhanced abdominal computed tomography scans at Dr George Mukhari Academic Hospital. South Afr J Radiol 25(1). 10.4102/sajr.v25i1.1990 PMC7876974 33604070

[8] Triantafyllou G, Belimezakis N, Lyros O, Węgiel A, Arkadopoulos N, Olewnik Ł, Tsakotos G, Zielinska N, Piagkou M (2025) Prevalence of coeliac trunk variants: A systematic review with meta-analysis. Annals Anat - Anatomischer Anzeiger 259:152385. 10.1016/j.aanat.2025.152385 39892002

[9] Arif F, Muhammad S, Khan MA, Khan AW (2021) Surgical experience of hepatectomy in a live donor with absent celiac trunk - a case report. Egypt Liver J 11(1). 10.1186/s43066-021-00107-6

[10] Guglielmo N, Meniconi RL, Vennarecci G, Ettorre GM (2020) Celiaco-mesenteric trunk: A rare variation that must be known before liver transplant. Dig Liver Disease 52(3):354. 10.1016/j.dld.2019.11.003 31982347

[11] Marcondes GB, Tenorio ER, Lima GB, Mendes B, Saqib N, Khan S et al (2021) Incorporation of Celiomesenteric Trunk With Double Kissing Directional Branches During Fenestrated-Branched Endovascular Aortic Repair. J Endovasc Ther 28(4):636–641. 10.1177/15266028211016430 33998350

[12] Koshariya M, Khare V, Songra MC, Shukla S, Gupta A (2021) Anomalous Anatomical Variations of Coeliac Trunk: A Cadaveric Study. Cureus. 10.7759/cureus.19108 PMC8627549 34868759

[13] Panagouli E, Lolis E, Venieratos D (2011) A morphometric study concerning the branching points of the main arteries in humans: Relationships and correlations. Annals Anat 193(2):86–99. 10.1016/j.aanat.2010.10.009 21169000

[14] Douard R, Chevallier JM, Delmas V, Cugnenc PH (2006) Clinical interest of digestive arterial trunk anastomoses. Surg Radiol Anat 28:219–227. 10.1007/s00276-006-0098-8 16547602

[15] Iliyas B, Nurlanbaev Y, Nagasbekov M, Kaniyev S, Baimakhanov B, Fakhradiyev I (2025) Distal pancreatectomy with celiac axis resection (DP-CAR) and vascular reconstruction for locally advanced pancreatic adenocarcinoma: a case report. Int J Surg Case Rep 130. 10.1016/j.ijscr.2025.111264 PMC12001092 40188696

[16] Latona JA, Lamb KM, Pucci MJ, Maley WR, Yeo CJ (2016) Modified Appleby Procedure with Arterial Reconstruction for Locally Advanced Pancreatic Adenocarcinoma: A Literature Review and Report of Three Unusual Cases. J Gastrointest Surg 20(2):300–306. 10.1007/s11605-015-3001-2 26525205

[17] Goto T, Fujimura H, Shintani T, Shibuya T, Miyagawa S (2024) Use of selective visceral angiography in surgical strategy planning for celiac artery aneurysm in the celiacomesenteric trunk. J Cardiothorac Surg 19(1). 10.1186/s13019-024-02483-7 PMC10797990 38243244

[18] Clark TWI (2006) Complications of hepatic chemoembolization. Semin Interv Radiol 23:119–125. 10.1055/s-2006-941442 PMC3036366 21326755

[19] Chaiwatcharayut W, Lekah A, Kurklinsky A (2013) Celiacomesenteric Trunk as a Cause of Median Arcuate Ligament Syndrome. J Med Ultrasound 21(1):39–42. 10.1016/j.jmu.2013.01.004

[20] Emekli E, Bayav FD, Gündoğdu E (2024) Exploring celiac trunk parameters in median arcuate ligament syndrome: A CT study. Surg Radiol Anat 46(6):805–810. 10.1007/s00276-024-03352-7 38622333

[21] Ganapathy A, Mohakud S, Rout S, Joy P, Alagappan A, Manokaran A (2024) A radio-anatomical study of median arcuate ligament syndrome: unveiling the morphology and morphometry of median arcuate ligament, celiac trunk, and superior mesenteric artery. Abdom Radiol 49(9):3297–3308. 10.1007/s00261-024-04231-w 38494467

[22] Tandler J (1904) Über die Varietäten der Arteria coeliaca und deren Entwickelung. Anatomische Hefte 25(2):473–500. 10.1007/BF02300762

[23] Higgins JPT, Thomas J, Chandler J, Cumpston M, Li T, Page MJ (2024) Cochrane Handbook for systematic reviews of interventions. Higgins JPT, Thomas J, Chandler J, Cumpston M, Li T, Page MJ (Eds). Co.

[24] Acar G (2022) Anatomical Variations of the Celiac Trunk and Hepatic Arteries with a New Classification, Based on CT Angiography. J Coll Physicians Surg Pakistan 32(1):9–14. 10.29271/jcpsp.2022.01.9 34983140

[25] Dillibabu E, Srinivasan K, Venkatraman I, Baskaran V, Komalavalli S, Sripriya S, Rajesh R, Mettu S, Reddy., Poonam KJ, Immanuel J, Pandiaraj, Niranjan Dhanaji K (2022) Newly proposed classification of celiac artery variations based on embryology and correlation with computed tomography angiography. Pol J Radiol 87:e563–e573. 10.5114/pjr.2022.120525 PMC9673974 36420123

[26] Mao Y, Yao Y, Li X, Zhang C, Chen X, Wang Y (2022) Absence of the celiac trunk: Definition, classification, multidetector computed tomography angiographic findings, and their probable embryological mechanisms. Vascular 31(6):1214–1221. 10.1177/17085381221106318 35634715

[27] Odabaşıoğlu ME, Cihan ÖF, Yılmaz MT (2021) Investigation of the Coeliac Trunk Morphometry with Multidetector Computed Tomography Angiography. Eur J Ther 27(1):55–65. 10.5152/eurjther.2021.20091

[28] Song S-Y, Chung JW, Yin YH, Jae HJ, Kim H-C, Jeon UB, Cho BH, So YH, Park JH (2010) Celiac Axis and Common Hepatic Artery Variations in 5002 Patients: Systematic Analysis with Spiral CT and DSA. Radiology 255(1):278–288. 10.1148/radiol.09090389 20308464

[29] Türkyılmaz Z, Kula O, Çelik AO, Demirel T, Günay B (2023) Evaluation of celiac artery and common hepatic artery variations by CT-angiography and new classification model. Surg Radiol Anat 45(8):1037–1047. 10.1007/s00276-023-03180-1 37340150

[30] Whitley A, Oliverius M, Kocián P, Havlůj L, Gürlich R, Kachlík D (2020) Variations of the celiac trunk investigated by multidetector computed tomography: Systematic review and meta-analysis with clinical correlations. Clin Anat 33(8):1249–1262. 10.1002/ca.23576 32012339

[31] Adachi B (1932) Das Arteriensystem der Japaner. In: Verlag der Kaiserlich-Japanischen Universität (Vol 66, pp 434–435). 10.1007/BF01493026

[32] Eaton PB (1917) The coeliac axis. Anat Rec 13(6):369–374. 10.1002/ar.1090130605

[33] Michels N (1955) Blood supply and anatomy of the upper abdominal organs. With a Descriptive Atlas. Br J Surg 43(181):560–560. 10.1002/bjs.18004318131

[34] Nateniyom N, Huanmanop T, Agthong S, Chentanez V (2019) Anatomy of the vasculature supplying hepatobiliary structures and celiac trunk branching patterns in the Thai population. Asian Biomed 12:161–167. 10.1515/abm-2019-0016

[35] Nelson TM, Pollak R, Jonasson O, Abcarian H (1988) Anatomic variants of the celiac, superior mesenteric, and inferior mesenteric arteries and their clinical relevance. Clin anatomy: official J Am association Clin anatomists Br association Clin anatomists 1(2):75–91. 10.1002/ca.980010202

[36] Vandamme JPJ, Bonte J (1980) The Branches of the Celiac Trunk. Acta Anat 122(2):110–114. 10.1159/000145991 4013640

[37] Marco-Clement I, Martinez-Barco A, Ahumada N, Simon C, Valderrama JM, Sanudo J, Arrazola J (2016) Anatomical variations of the celiac trunk: cadaveric and radiological study. Surg Radiol Anat 38(4):501–510. 10.1007/s00276-015-1542-4 26267305

[38] Panagouli E, Venieratos D, Lolis E, Skandalakis P (2013) Variations in the anatomy of the celiac trunk: A systematic review and clinical implications. Annals Anatomy-Anatomischer Anzeiger 195(6):501–511. 10.1016/j.aanat.2013.06.003 23972701

[39] Lippert H, Pabst R (1985) Arterial variations in man: classification and frequency. München: JF Bergmann. 10.1001/jama.1986.03370200123049

[40] Morita M (1935) Reports and conception of three anomalous cases on the area of the celiac and the superior mesenteric arteries. Igaku Kenkyu 9:1993–2006. https://cir.nii.ac.jp/crid/1573105974092542080

[41] Uflacker R (2007) Atlas of vascular anatomy: an angiographic approach

